# A latent profile analysis of the pain-fatigue-sleep disturbance symptom clusters in postoperative cervical cancer patients: a multicenter cross-sectional study

**DOI:** 10.3389/fpubh.2026.1901928

**Published:** 2026-08-26

**Authors:** Kaiyi Wang, Xinru Ma, Huan Wang

**Affiliations:** School of Nursing, Jinzhou Medical University, Jinzhou, Liaoning, China

**Keywords:** cervical cancer, influencing factors, latent profile analysis, postoperative, symptom clusters

## Abstract

**Objective:**

To investigate the potential subtypes and influencing factors of the pain-fatigue-sleep disturbance (PFS) symptom cluster in postoperative cervical cancer patients, thereby providing a basis for developing targeted intervention measures.

**Methods:**

A cross-sectional study design was adopted. Using convenience sampling, 374 postoperative cervical cancer patients admitted to three Grade A Level 3 hospitals in Liaoning Province between April 2025 and September 2025 were selected as study subjects. Questionnaire surveys were conducted using a demographic information form, the Brief Pain Scale, the Fatigue Severity Scale, the Pittsburgh Sleep Quality Index, the Hospital Depression and Anxiety Scale, and the Perceived Social Support Scale. Model fit indices for Latent Profile Analysis (LPA) included the Akaike Information Criterion (AIC), Bayesian Information Criterion (BIC), sample-corrected Bayesian Information Criterion (aBIC), entropy, the Lore-Mundel-Rubin-Tobin (LMRT) test, and the bootstrap-based likelihood ratio test (BLRT). The AIC, BIC, and aBIC values of the four-profile model were markedly lower, indicating a significant improvement in model fit. Both the LMRT and BLRT test results showed *p* < 0.001, confirming that the four-category classification did not merely improve model fit by increasing the number of categories; rather, there are objectively four distinct patterns of pain-fatigue-sleep disturbance symptom combinations. The entropy value of 0.848 indicates excellent discriminatory ability among the subtypes, with nearly all patients accurately classified into their corresponding symptom subtypes. The study also identified influencing factors through univariate and multivariate logistic regression analyses.

**Results:**

A total of 374 valid questionnaires were collected, with a response rate of 95.9%. The PFS of postoperative cervical cancer patients can be classified into four potential categories: Low Symptom Expression Type (29.679%), Pain-Dominated—Sleep-Impaired Type (26.738%), High Pain and Fatigue—Sleep Compensation Type (26.471%), and Multisymptom Coexistence—Sleep Disorder Type (17.112%). Logistic regression analysis revealed that perceived social support and laparoscopic surgery were common protective factors; hospital-related anxiety and depression were common risk factors; monthly household income, age, place of residence, postoperative complications, and occupation were specific risk factors.

**Conclusion:**

There is population heterogeneity in PFS among postoperative cervical cancer patients. Healthcare providers should implement targeted interventions based on protective and risk factors specific to different latent profiles to promote effective management of this symptom cluster.

## 1 Introduction

Cervical cancer is the fourth most common cancer among women globally. In 2022, the global number of new cervical cancer cases reached 661,000, leading to 348,000 deaths (1), and most of these cases and deaths occurred in developing regions (2). Cervical cancer is the leading cause of cancer incidence among women in approximately 13% of countries worldwide, and it is the primary cause of cancer-related mortality in nearly 37 countries (3). This substantial disease burden constitutes a severe challenge to global public health systems, especially for cancer prevention, control, and rehabilitation services in resource-constrained regions.

For patients with International Federation of Gynecology and Obstetrics (FIGO) stage IA2 to IIA2 cervical cancer, radical hysterectomy is the primary treatment modality (4), and the 5-year survival rate for patients with early-stage cervical cancer exceeds 95% (5).However, surgery often leads to various complications that significantly impact recovery and quality of life. The incidence of cancer-related fatigue reaches as high as 50%−70% within 2 months post-surgery (6), and approximately 40%−65% of patients continue to experience moderate to severe pain 4 to 8 weeks after surgery (7). The severity of these symptoms is closely associated with anxiety, depression, and sleep disturbances (8). Among patients with gynecologic cancers, a proven association exists between sleep disturbances and postoperative pain (9). From a public health perspective, these symptoms not only reduce individual patients' quality of life but may also increase unnecessary use of health services and care costs, highlighting the urgency of optimizing postoperative symptom management.

Fatigue, pain, and sleep disturbances are common co-occurring symptoms (10, 11); they often interact to form a “symptom cluster,” exacerbating patients' suffering and complicating symptom management. Existing research confirms that PFS symptom clusters exhibit high heterogeneity and persistence among survivors of various malignancies, including lung cancer (12), breast cancer (13), and colorectal cancer (14). Their negative impact extends beyond the simple sum of individual symptoms, forming a complex pathophysiological network that leads to long-term functional limitations and economic toxicity. Furthermore, studies (15) indicate that patients with specific cancer types, such as head and neck cancer, may face severe treatment-related financial stress that can even lead to bankruptcy, while the presence of symptom clusters such as PFS increases the need for symptomatic supportive care, further driving up direct medical costs. However, the characteristics and patterns of this symptom cluster in postoperative cervical cancer patients remain unclear. In multi-symptom cluster studies targeting the general gynecologic oncology population, a small number of studies have included mixed samples of cervical, ovarian, and endometrial cancer patients, identifying composite symptom clusters comprising hot flashes, urinary symptoms, and pain (16). However, most studies have combined cervical cancer patients with those with ovarian and endometrial cancers in their analyses, lacking a specific exploration of symptom cluster stratification tailored to postoperative cervical cancer patients. Pain, fatigue, and sleep disturbances constitute the group of physical symptoms with the highest incidence and the closest interrelationship during the short-term recovery phase following cervical cancer surgery (17). Compared to symptoms such as pelvic floor dysfunction and hot flashes—which are highly correlated with the extent of surgery and whether the ovaries are preserved—PFS is present in nearly all surgical patients and is therefore more universal. While other postoperative symptoms, such as urinary incontinence and lymphedema, do affect daily activities, they rarely directly and persistently disrupt circadian rhythms and overall energy levels, and thus have a weaker dual physical and psychological impact on patients than the PFS cluster (18). Furthermore, existing studies primarily focus on average symptom levels, treating patients as a homogeneous group. This approach obscures potential subtypes with distinct symptom patterns, thereby limiting the development of precision intervention strategies and hindering the efficient allocation of public health resources for symptom management.

Latent Profile Analysis (LPA), as an individual-centered statistical method capable of classifying individuals into different latent profiles based on their response patterns across multiple variables, has been successfully applied to identify symptom subtypes among patients with other types of cancer (12, 13). Existing research has confirmed that the PFS symptom cluster is prevalent among cancer patients and that these symptoms interact with one another; however, three key research gaps exist in this field: first, although the PFS symptom cluster has been verified in multiple malignancies„ including breast cancer (19) and lung cancer (20), no prior study has profiled the three-symptom PFS triad among postoperative cervical cancer patients; second, existing studies (21) have inherent limitations in their perspective: they treat all postoperative cervical cancer patients as a homogeneous group with consistent symptom characteristics, overlooking the heterogeneity within the population. This fails to explain the phenomenon observed in clinical practice, where, even after receiving the same surgery and nursing interventions, patients still exhibit significant differences in symptom severity and patterns, thereby hindering the development of precision-tailored, stratified rehabilitation intervention plans; Third, existing discussions on the determinants of postoperative cervical cancer patients are largely confined to clinical indicators such as surgical approach, pathological staging, and postoperative complications. Rarely do they integrate health and social determinants—such as household income, place of residence, employment status, and social support—to analyze intergroup differences in symptom clusters. The lack of stratified research on postoperative symptom subtypes from the perspective of health and social inequalities also makes it difficult to develop public health intervention strategies that take equity into account.

Addressing these three research gaps, this study offers significant novelty in three areas: methodology, research content, and public health application. First, at the methodological level, it is the first to employ the individual-centered statistical method LPA to objectively stratify PFS symptom clusters in postoperative cervical cancer patients, overcoming the limitations of traditional mean-based variable analysis and accurately identifying heterogeneous symptom subtypes; Second, in terms of research content, this study simultaneously incorporates multidimensional variables—including clinical indicators, sociodemographic characteristics, psychological and emotional factors, and social support—to distinguish between protective and risk factors common to all subtypes and those specific to each subtype, thereby addressing the shortcoming of previous studies that analyzed only the independent risk factors for individual symptoms; Third, at the public health application level, grounded in the perspective of health inequalities, the study reveals the differential effects of social factors—such as urban versus rural residence, household income, and employment status—on different symptom subtypes, providing evidence for stratified, equity-focused individualized interventions and filling the gap of empirical data on population-stratified interventions in cancer rehabilitation public health research.

## 2 Materials and methods

### 2.1 Study design

Convenience sampling was employed. Between April and September 2025, cervical cancer patients seeking treatment at three Grade A Level 3 hospitals in Liaoning Province were selected as study participants.

### 2.2 Participants and sample

**Figure 1:**
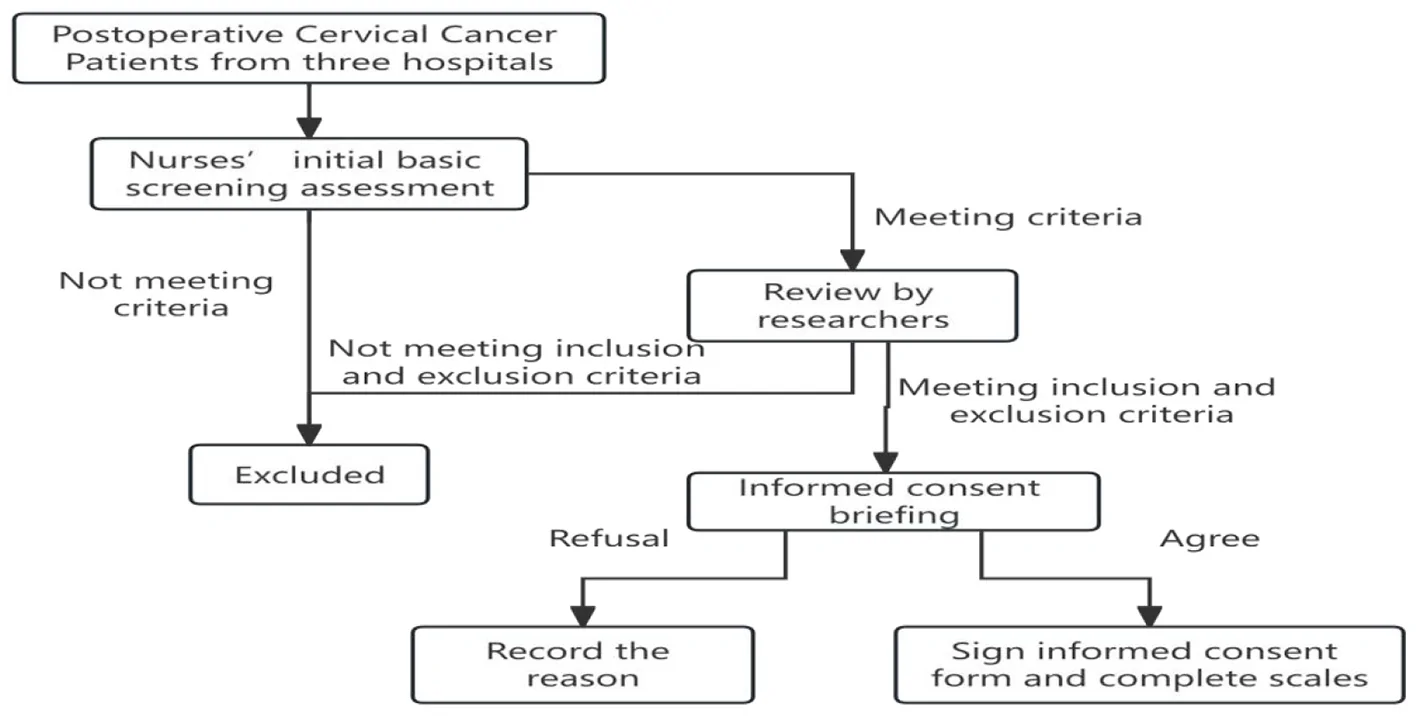
Flowchart of patient screening and enrollment.

Inclusion Criteria: ① Patients with a first-time diagnosis of primary cervical cancer confirmed by histopathological examination (22) ② Patients undergoing surgical treatment for the first time ③ Age ≥ 18 years ④ Within 2 months post-surgery ⑤ Patients who are alert, oriented, and not currently receiving adjuvant radiotherapy or chemotherapy ⑥ Patients who are alert and possess full verbal communication abilities ⑦ Patients who have provided informed consent and voluntarily participated in this study. Exclusion Criteria: ① Concurrent primary malignant tumors; ② Diagnosis of severe psychiatric disorders (e.g., schizophrenia, bipolar disorder, etc.); ③ Insufficient function of vital organs such as the heart or lungs; ④ Participation in other interventional clinical trials. Based on Kendall's sample size estimation method (23), the sample size for quantitative research should be 5–10 times the number of variables. Taking into account a 20% rate of invalid questionnaires, the sample size formula for this study is *N* = 27 × (5–10)/(1–20%) = 169–338; a total of 374 patients were finally recruited. This study was conducted at three Class A Grade III hospitals in Liaoning Province, with a recruitment period from April 2025 to September 2025. The standardized recruitment process was as follows: first, ward nurses conducted daily preliminary screenings; second, research investigators reviewed all inclusion and exclusion criteria; subsequently, patients were fully informed about the study and signed written informed consent forms; guiding patients to complete all questionnaires on-site; upon completion, the questionnaire information was verified immediately, and any omissions or errors were corrected; finally, valid questionnaires were uniformly numbered and archived. For subject eligibility screening, ward nurses first conducted a basic preliminary screening based on postoperative duration, surgical history, and age, followed by researchers verifying each inclusion and exclusion criterion item by item; subjects failing to meet any criterion were immediately excluded; patients who met all criteria were formally enrolled in this study after completing the informed consent process and signing the consent form see the process flow in the Figure 1. This study has been approved by the hospital's Institutional Review Board (IRB Approval No.: JZMULL2025358), and all participants provided informed consent and participated voluntarily.

### 2.3 Sociodemographic and clinical characteristics

The researchers designed the data collection framework, which included age, marital status, educational level, occupation, monthly household income, place of residence, payment method, pathological diagnosis, surgical procedure, time since surgery, pathological stage, and postoperative complications.

### 2.4 Fatigue

The Fatigue Severity Scale (FSS) was developed in 1989 by American researchers Krupp et al. (24) through clinical observations and was first introduced to China by Wu (25). The scale consists of nine items, each using a 7-point Likert scale ranging from 1 (“Strongly Disagree”) to 7 (“Strongly Agree”). The FSS score is calculated as the mean of the nine items (total score/9); a higher score indicates a higher level of fatigue (FSS score ≤ 4 = no fatigue, 4.1–4.9 = suspected fatigue, and ≥ 5 = clinical fatigue.) In this study, the Cronbach's α coefficient for this scale was 0.864.

### 2.5 Pain

The Brief Pain Inventory (BPI) was developed in the late 1980s under the leadership of Professor Cleeland (26) and translated by Wang et al. (27).This scale systematically assesses the severity of pain and its impact on daily functioning. It covers two dimensions—pain intensity and pain impact—and consists of 17 items. A score of 0 indicates no impact, 1–3 indicates mild impact, 4–6 indicates moderate impact, 7–9 indicates severe impact, and 10 indicates complete impact. In this study, the Cronbach's α coefficient for this scale was 0.922.

### 2.6 Sleep disorders

The Pittsburgh Sleep Quality Index (PSQI) was developed in 1989 by Buysse et al. (28) at the University of Pittsburgh in the United States and adapted into Chinese by Liu (29) and colleagues. The scale covers seven dimensions, including subjective sleep quality, sleep latency, and sleep duration, and consists of 18 items in total. The scoring range for each dimension is 0–3 points, with a total score ranging from 0 to 21 points. A score of ≤ 7 indicates good sleep quality; a score >7 suggests the presence of a sleep disorder. In this study, the Cronbach's α coefficient for this scale was 0.793.

### 2.7 Anxiety and depression

The Hospital Anxiety and Depression Scale (HADS) was developed by Zigmond et al. in 1983 (30), with the aim of screening for nonpsychotic anxiety and depressive symptoms among hospitalized patients. The scale consists of two independent subscales, both of which use a 4-point Likert scale for scoring, with scores ranging from 0 to 3. The total score for each subscale ranges from 0 to 21; a higher score indicates a more severe emotional response in the patient. A total score of 0 to 7 indicates no symptoms of anxiety or depression; 8 to 10 indicates a possible tendency toward anxiety or depression; and 11 to 21 indicates relatively significant symptoms. With 8 as the cutoff point, a score of ≥8 is considered a positive result. In this study, the Cronbach's α coefficient for this scale was 0.890.

### 2.8 Social support

The Perceived Social Support Scale (PSSS) was developed by Zimet et al.^.^(31) and was translated into Chinese in 2001 by Fan (32). The scale includes three dimensions—family, friends, and other sources of support—comprising a total of 12 items. It uses a 1–7-point rating scale, where 1 indicates “strongly disagree” and 7 indicates “strongly agree,” with a total score range of 12–84. A higher score indicates a higher level of perceived social support. In this study, the Cronbach's α coefficient for this scale was 0.899.

### 2.9 Data analysis

SPSS 27.0 [IBM Corporation (SPSS is an IBM Company), Armonk, NY, USA] was used for descriptive statistics, univariate analysis, and multivariate logistic regression analysis, while Mplus 8.3 (Muthén & Muthén, Los Angeles, CA, USA) was used for LPA. All statistical tests were two-sided, with a significance level of α = 0.05; *p* < 0.05 was considered statistically significant. Continuous variables are expressed as the mean ± standard deviation, and comparisons between groups are performed using analysis of variance (ANOVA); categorical data are expressed as frequencies and percentages, and comparisons between groups are performed using the χ^2^test. Model fit indices for LPA included the Akaike Information Criterion (AIC), Bayesian Information Criterion (BIC), sample-corrected Bayesian Information Criterion (aBIC), entropy, the Lore-Mundel-Rubin-Tobin (LMRT) test, and the bootstrap-based likelihood ratio test (BLRT). Among these, smaller AIC, BIC, and aBIC values indicate better model fit; the closer the entropy value is to 1, the higher the classification accuracy. The LMRT and BLRT are used to compare the differences in goodness of fit between K-category models and (K−1)-category models; when *p* < 0.05, the K-category model provides a better fit. According to methodological consensus, the sample proportion for all potential subtypes should be no less than 5% of the total sample size to avoid sampling bias caused by extremely small categories. In this study, the four subtypes identified by the 4-category model accounted for 29.679%, 26.738%, 26.471%, and 17.112% of the total, respectively—all above the 5% threshold. No subtypes with an excessively low sample proportion were identified; each subtype had an adequate sample size and exhibited a balanced and stable distribution. Regarding model stability testing, the four-category profile model was refitted multiple times in Mplus using different random seeds. The classification agreement for patients in each subtype was higher than 0.85, the entropy value remained stable at around 0.848, and the results of the LMRT and BLRT tests were consistent across repetitions. In contrast, after multiple refits, the category boundaries in the three- and five-category models fluctuated significantly, and the groupings were unstable, demonstrating that the results of the four-category model are reliable and reproducible. From a clinical practice perspective, the three-category model can only roughly distinguish between patients with mild, moderate, and severe symptoms, making it difficult to support the development of precise nursing care plans; the five-category model, on the other hand, over-segments the population, significantly increasing the workload for clinical nurses in stratified assessment and care, and thus lacks practical applicability for widespread implementation. The four-category classification aligns perfectly with the clinical rehabilitation context following cervical cancer surgery and can directly guide stratified nursing interventions, demonstrating strong clinical applicability. Comprehensive statistical analysis ultimately confirmed the four-category profile as the optimal solution. A multivariate logistic regression analysis was conducted using variables with statistical significance in the univariate analysis as independent variables, and the fitted latent profiles as dependent variables, with a significance level of α = 0.05. Prior to modeling, a multicollinearity diagnosis was conducted on the included independent variables using tolerance, the variance inflation factor (VIF) and the condition index to test for multicollinearity; severe multicollinearity was defined as VIF > 10 or the condition index > 30 (33). Upon completion of modeling, a multi-criteria comprehensive evaluation model was employed to assess fit quality: overall model validity was determined using the multinomial logistic regression model and the Pearson chi-square test, with the Deviance test serving as the primary criterion; Cox-Snell, Nagelkerke, and McFadden pseudo-*R*^2^ values were utilized to reflect model explanatory power; AIC and BIC information criteria were incorporated to assist in model performance assessment; and univariate likelihood ratio tests were applied to identify independent predictors for symptom subtypes. Since the four latent symptom subtypes as the dependent variable have no inherent severity ranking, unordered multinomial logistic regression was adopted instead of ordinal logistic regression. The proportional hazards assumption, inherent to ordered logistic regression, was not met in this study; consequently, no proportional hazards homogeneity test was conducted. There were no missing data in this study, and outliers were excluded through questionnaire quality control.

## 3 Results

### 3.1 LPA results

This study used the FSS total score, BPI dimension scores, and PSQI total score as manifest variables. Starting with a latent profile model with one category, the number of categories was gradually increased to identify the optimal classification model. The fit indices and distribution characteristics corresponding to different numbers of categories are shown in Table 1. The optimal model was selected through a stepwise screening process that considered statistical parameters, subtype size, and clinical interpretability: the 2-category model exhibited significantly higher AIC, BIC, and aBIC values, indicating limited model optimization. It could only distinguish between mild and severe groups and failed to differentiate patient subtypes dominated by pain, fatigue, or sleep disturbances, thereby failing to support stratified care that identifies subtypes with distinct symptom dominance; the 3-category model performed worse overall than the 4-category model across all information criteria; its grouping was too broad, combining patients with prominent pain and fatigue but acceptable sleep with those experiencing simultaneous worsening of multiple symptoms, thereby losing key symptom heterogeneity; although the 5-class model has the lowest information criteria values, its fit improvement relative to the 4-class model is significantly reduced; the LMRT test *p*-value decreases, entropy decreases, and classification accuracy declines. Furthermore, it includes subtypes with a small sample proportion, and the newly added groups lack distinct clinical characteristics. In contrast, while the 4-class model shows a substantial decrease in information criteria compared to the 2- and 3-class models, the test results confirm a highly significant improvement in fit. It exhibits good entropy values and balanced, stable sample distributions across all subtypes. The four classifications accurately correspond to different symptom burden groups among postoperative cervical cancer patients. combining both statistical performance and clinical value; therefore, the 4-class classification was ultimately determined to be the optimal model.

**Table 1:** Latent profile analysis indicators for PFS in postoperative cervical cancer patients (*n* = 374).

Model	AIC	BIC	Abic	Information Entropy	LMRT (*p*)	BLRT (*p*)	Category probability (%)
1	4,257.459	4,288.853	4,263.471	—	—	—	—
2	3,606.998	3,658.013	3,616.768	0.875	0.0000	0.0000	60.695/39.305
3	3,326.271	3,396.907	3,339.798	0.874	0.0007	0.0000	39.305/40.909/19.786
4	3,230.723	3,320.981	3,248.008	0.848	0.0000	0.0000	29.679/26.738/26.471/17.112
5	3,206.477	3,316.356	3,227.520	0.836	0.0415	0.0000	18.449/13.369/28.075/16.310/23.797

**Figure 2:**
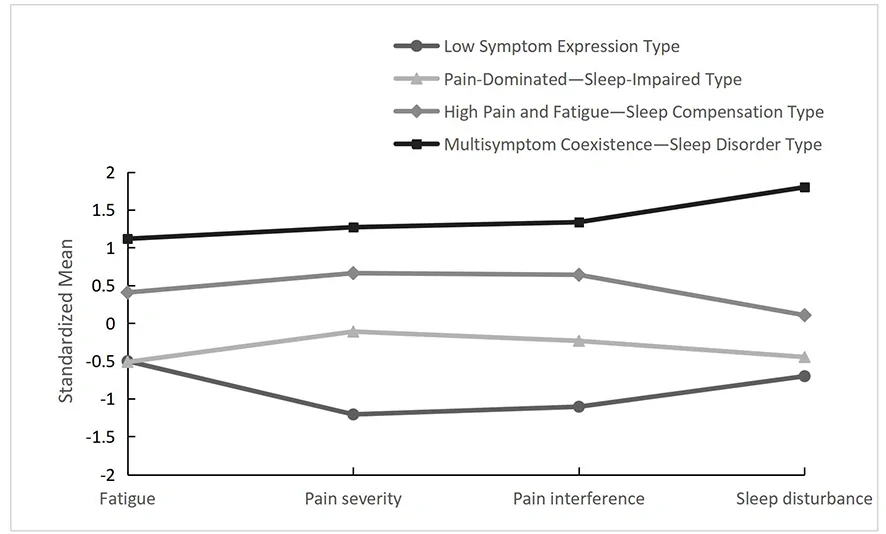
Standardized mean scores of symptom dimensions across the four latent profiles.

Figure 2 shows the distribution of mean scores across the four subtypes after symptom standardization. Group C1 scored negatively across all dimensions—fatigue, pain severity, pain distress, and sleep disturbance (−0.502, −1.207, −1.105, and −0.701, respectively). The severity of negativity was consistent across all dimensions, with no single symptom dimension standing out; this group was named the “Low Symptom Expression Type.” From a statistical perspective, this group showed no significant score deviations in any of the pain, fatigue, or sleep dimensions, had no predominant symptom, and exhibited a relatively low overall symptom burden. Clinically, there is no need to implement intensive analgesia, anti-fatigue, or specialized sleep interventions; “Low Symptom Expression” accurately summarizes the core characteristics of this subtype. Group C2 also had negative scores across all symptom dimensions, but pain severity was significantly higher than in Group C1; this subtype was named the “Pain-Dominated—Sleep-Impaired Type.” In this group, only the severity of pain and pain-related distress were relatively prominent, distinguishing it from the multisymptom severe group; clinical intervention should focus on pain relief, with sleep requiring only basic supportive care. The term “pain-dominant” in the name clarifies the primary intervention target for this subtype, while “impaired sleep” clearly distinguishes it from the severe insomnia subtype. Group C3 had positive scores across all symptom dimensions, with pain and fatigue scores significantly higher than those for sleep disturbances; although sleep disturbances were above the overall average, the increase was lower than that of the former two. This group was named the “High Pain and Fatigue—Sleep Compensation Type.” In this group, pain and fatigue are the core symptoms with high scores; although sleep disturbances have worsened, the rate of increase is far lower than that of the former two. This classification is based solely on the distribution of symptom scores from the cross-sectional data in this study and serves only to objectively describe the external symptoms of this patient group, characterized by prominent pain and fatigue but relatively mild sleep disturbances. Pain and fatigue are the core symptoms in this group, and although sleep disturbances are present, their overall severity is mild. Existing research (34) indicates that when moderate-to-severe pain coexists with fatigue, the body may activate compensation sleep regulation mechanisms, relying on extended sleep duration and deeper sleep to alleviate physical damage and inflammatory stress. However, this study collected only subjective self-report data from patients; there is no objective sleep monitoring, inflammatory markers, or endocrine physiological indicators to corroborate the actual existence of this compensation pathway. Therefore, this finding is provided solely as a reference for interpreting the phenomenon and does not constitute a definitive conclusion regarding the underlying mechanism. All symptom dimension scores in Group C4 were high positive values, with the most pronounced increase observed in the sleep disturbance dimension; this subtype was named “Multisymptom Coexistence—Sleep Disorder Type.”. In this subtype, fatigue, pain, and sleep disturbances all increase severely in tandem, with no clear hierarchy among the three symptom categories; patients simultaneously endure multiple severe physical distressors.

### 3.2 Patient demographics and results of univariate analysis

A total of 390 questionnaires were distributed, and 374 valid questionnaires were recovered, yielding an effective response rate of 95.9%. The mean HADS score of the 374 postoperative cervical cancer patients was 10.38 ± 6.57, and the FSS scores were 30.03 ± 11.49 points. Other general demographic data are presented in Table 2.

**Table 2:** General characteristics of postoperative cervical cancer patients and univariate analysis of potential PFS profiles (*n* = 374).

Item	Total	Low symptom expression type (n = 111)	Pain-dominated—Sleep-impaired type (*n* = 100)	High pain and fatigue—sleep compensation type (*n* = 99)	Multisymptom coexistence—sleep disorder type (*n* = 64)	Test statistic	*p-value*
**Age**						114.652^a^	< 0.001
18–40 years	157 (42.0)	53 (47.7)	80 (80.0)	15 (15.2)	9 (14.1)		
Ages 41–59	143 (38.2)	39 (35.1)	18 (18.0)	50 (50.5)	36 (56.3)		
60 years and older	74 (19.8)	19 (17.1)	2 (2.0)	34 (34.3)	19 (29.7)		
Marital status						6.321^a^	0.388
Married	260 (69.5)	75 (67.7)	74 (74.0)	70 (70.7)	41 (64.1)		
Unmarried	53 (14.2)	16 (14.4)	8 (8.0)	16 (16.2)	13 (20.3)		
Other	61 (16.3)	20 (18.0)	18 (18.0)	13 (13.1)	10 (15.6)		
Educational level						9.927^a^	0.128
Junior high school and below	145 (38.8)	40 (36.0)	36 (36.0)	43 (43.4)	26 (40.6)		
High school/vocational school	173 (46.3)	52 (46.8)	51 (51.0)	36 (36.4)	34 (53.1)		
College degree or higher	56 (15.0)	19 (17.1)	13 (13.0)	20 (20.2)	4 (6.3)		
Occupation						28.534^a^	0.001
Employed	168 (44.9)	45 (40.5)	56 (56.0)	48 (48.5)	19 (29.7)		
Farmers	94 (25.1)	35 (31.5)	24 (24.0)	23 (23.2)	12 (18.8)		
Unemployed	71 (19.0)	18 (16.2)	9 (9.0)	20 (20.2)	24 (37.5)		
Other	41 (11.0)	13 (11.7)	11 (11.0)	8 (8.1)	9 (14.1)		
Monthly household income						61.138^a^	< 0.001
< 5,000 yuan	145 (38.8)	32 (28.8)	17 (17.0)	53 (53.5)	43 (67.2)		
5,000–10,000 yuan	141 (37.7)	53 (47.7)	44 (44.0)	28 (28.3)	16 (25.0)		
10,000 or more	88 (23.5)	26 (23.4)	39 (39.0)	18 (18.2)	5 (7.8)		
Place of residence						23.434^a^	< 0.001
City	198 (52.9)	62 (55.9)	64 (64.0)	55 (55.6)	17 (26.6)		
Rural	176 (47.1)	49 (44.1)	36 (36.0)	44 (44.4)	47 (73.4)		
Payment Method						3.784^a^	0.706
Self-pay	101 (27.0)	26 (23.4)	27 (27.0)	32 (32.3)	16 (25.0)		
Health insurance	218 (58.3)	70 (63.1)	60 (60.0)	52 (52.5)	36 (56.3)		
Other	55 (14.7)	15 (13.5)	13 (13.0)	15 (15.2)	12 (18.8)		
Pathological diagnosis						2.916^a^	0.819
Cervical squamous cell carcinoma	306 (81.8)	93 (83.8)	80 (80.0)	81 (81.8)	52 (81.3)		
Cervical adenocarcinoma	41 (11.0)	9 (8.1)	11 (11.0)	12 (12.1)	9 (14.1)		
Other	27 (7.2)	9 (8.1)	9 (9.0)	6 (6.1)	3 (4.7)		
Surgical Method						26.508^a^	< 0.001
Open surgery	191 (51.1)	35 (31.5)	65 (65.0)	57 (57.6)	34 (53.1)		
Laparoscopy	183 (48.9)	76 (68.5)	35 (35.0)	42 (42.4)	30 (46.9)		
Postoperative time						4.191^a^	0.898
< 1 week	172 (46.0)	48 (43.2)	46 (46.0)	48 (48.5)	30 (46.9)		
2–4 weeks	115 (30.7)	33 (29.7)	34 (34.0)	30 (30.3)	18 (28.1)		
January–February	35 (9.4)	11 (9.9)	8 (8.0)	11 (11.1)	5 (7.8)		
> February	52 (13.9)	19 (17.1)	12 (12.0)	10 (10.1)	11 (17.2)		
Pathological Stage						10.861^a^	0.541
Stage I A1	77 (20.6)	24 (21.6)	19 (19.0)	26 (26.3)	8 (12.5)		
Stage I A2	42 (11.2)	9 (8.1)	14 (14.0)	13 (13.1)	6 (9.4)		
Stage IB	91 (24.3)	30 (27.0)	23 (23.0)	23 (23.2)	15 (23.4)		
Stage IIA	106 (28.3)	33 (29.7)	26 (26.0)	26 (26.3)	21 (32.8)		
Stage IIB and above	58 (15.5)	15 (13.5)	18 (18.0)	11 (11.1)	14 (21.9)		
Postoperative complications						76.150^a^	< 0.001
No	257 (68.7)	92 (82.9)	78 (78.0)	72 (72.7)	15 (23.4)		
Yes	117 (31.3)	19 (17.1)	22 (22.0)	27 (27.3)	49 (76.6)		
Perceived Social Support score	–	40.24 ± 10.59	30.69 ± 7.45	23.82 ± 7.69	20.89 ± 8.73	90.147^b^	< 0.001
Hospital anxiety and depression scale (HADS) score	–	4.703 ± 4.216	9.40 ± 4.82	13.12 ± 4.29	17.52 ± 6.15	112.152^b^	< 0.001

^a^represents the value of χ^2^; ^b^represents the F-value. Except for the Perceived Social Support Score and the Perceived Social Support Score, which are expressed as mean ± standard deviation, data for all other items are presented as frequency and percentage.

### 3.3 Multivariate logistic regression analysis of potential profiling factors

PFS subtype was used as the dependent variable, with the “Low Symptom Expression Type” (C1) serving as the reference group. This study included eight independent variables: age, occupation, monthly household income, place of residence, surgical procedure, postoperative complications, total perceived social support score, and HADS score. The tolerance values ranged from 0.546 to 0.892, VIF ranging from 1.122 to 1.831, and a maximum condition index of 4.539. None of these values reached the thresholds for multicollinearity, indicating no significant multicollinearity among the independent variables; thus, all variables could be included in the unordered multiclass logistic regression model. The fitting results of the unordered multiclass logistic regression model are as follows: Overall model likelihood ratio test χ^2^ = 473.401, df = 36, *p* < 0.001, indicating that the eight included independent variables as a whole can significantly predict the postoperative symptom classification in cervical cancer patients; the goodness-of-fit deviation test χ^2^ = 546.449, df = 1077, *p* = 1.000 > 0.005, suggesting that the model fits well; the Pearson chi-square test showed significance due to uneven cell distributions and is provided for reference only; the Nagelkerke pseudo *R*^2^ of the model was 0.768, indicating that the independent variables have strong explanatory power for symptom subtyping; The AIC and BIC values of the full model are significantly lower than those of the empty model, further validating its superior fit. The multiclass logistic regression results show that, with the low-symptom phenotype as the reference, perceived social support and laparoscopic surgery are common protective factors for patients; hospital-related anxiety and depression are common risk factors; and monthly household income, age, place of residence, postoperative complications, and occupation are specific risk factors. See Table 3 for details

**Table 3:** Results of unordered multi-class logistic regression analysis on potential factors influencing PFS in postoperative cervical cancer patients (*n* = 374).

Variable	Pain-dominated—sleep-impaired type				High pain and fatigue—sleep compensation type				Multisymptom coexistence—sleep disorder type			
	*β*	*P*-value	OR	95% CI	*β*	*P*-value	OR	95% CI	*β*	*P*-value	OR	95% CI
Constant	4.580	0.002	—	—	4.873	0.009	—	—	−5.335	0.030	—	—
Age												
41–59 years	−1.037	0.012	0.354	0.157–0.798	1.444	0.005	4.237	1.538–11.675	2.366	0.001	10.658	2.480–45.810
60 years or older	−1.920	0.023	0.147	0.028–0.764	2.354	0.001	10.524	2.607–42.488	2.953	0.001	19.161	3.107–118.172
Occupation												
Unemployed	0.493	0.455	1.637	0.449–5.971	0.442	0.552	1.556	0.363–6.668	1.949	0.028	7.024	1.234–39.981
Monthly household income												
5,000–10,000 yuan	−0.552	0.285	0.579	0.209–1.584	−1.758	0.004	0.172	0.052–0.574	−1.808	0.012	0.164	0.040–0.674
10,000 or more	−0.044	0.936	0.957	0.326–2.807	−1.365	0.040	0.255	0.069–0.939	−1.948	0.033	0.143	0.024–0.850
Place of residence												
Rural	−0.107	0.784	0.899	0.419–1.927	−0.016	0.974	0.984	0.388–2.499	1.635	0.011	5.129	1.463–17.974
Surgical approach												
Laparoscopic	−0.904	0.035	0.405	0.174–0.940	−1.075	0.038	0.341	0.124–0.941	−2.037	0.002	0.130	0.036–0.471
Postoperative complications	−0.814	0.067	0.443	0.185–1.059	−0.874	0.107	0.417	0.144–1.209	1.607	0.014	4.987	1.376–18.073
Perceived social support	−0.079	< 0.001	0.924	0.889–0.962	−0.152	< 0.001	0.859	0.813–0.907	−0.110	< 0.001	0.895	0.839–0.956
Hospital Anxiety and Depression	0.183	< 0.001	1.201	1.101–1.310	0.294	< 0.001	1.342	1.213–1.484	0.454	< 0.001	1.575	1.396–1.775

## 4 Discussion

### 4.1 Population heterogeneity in PFS among postoperative cervical cancer patients

This study used LPA to identify four distinct categories of PFS among postoperative cervical cancer patients, suggesting significant population heterogeneity in PFS. From a public health perspective, this heterogeneity implies that a one-size-fits-all symptom management approach may fail to effectively address the needs of different patient subtypes and could even exacerbate inequalities in recovery outcomes. ① The “Low Symptom Expression Type” subtype accounted for 29.679%, with patients in this group experiencing the lightest symptom burden. Laparoscopic surgery, as a minimally invasive treatment, causes minimal trauma to pelvic tissues and is associated with lower levels of postoperative pain and fatigue (35); simultaneously, patients without postoperative complications are more likely to exhibit mild symptom clusters. These two factors may explain the distribution characteristics of the relatively light symptom burden in this subtype. ② The “Pain-Dominated—Sleep-Impaired Type” subtype accounted for 26.738%. Younger patients had lower pain thresholds than older patients (36). Abdominal incisions from open surgery and extensive pelvic tissue damage were associated with persistent postoperative pain (37). Employed patients typically have a stable income; better financial circumstances may be associated with greater access to medical resources, and in this study, this was consistent with a trend toward relatively milder pain reports. ③ High Pain and Fatigue—Sleep Compensation Type accounted for 26.471%; middle-aged and older patients have relatively reduced tissue repair capacity, compounded by the significant trauma of open surgery; both factors are associated with higher levels of pain and fatigue symptoms; Individuals with lower household incomes have relatively limited access to high-quality rehabilitation support, which may be associated with persistent physical discomfort. This may be related to the fact that, under the dual stress of pain and fatigue, the body uses deep sleep to promote recovery and alleviate the burden of symptoms (34). ④ Multisymptom Coexistence—Sleep Disorder Type accounts for 17.112%. Postoperative follow-up care resources available to rural residents are relatively insufficient, and patients have limited ability to recognize and self-manage their symptoms; unemployment and low income often lead to significant financial pressure. Additionally, the incidence of postoperative complications in this subtype is as high as 76.6%. All of the above factors are associated with the overlap of multiple symptoms and severe sleep disturbances. A study (38) confirms that chronic physical pain is associated with sleep disturbances and abnormalities in the hypothalamic-pituitary-adrenal axis; together, these factors may exacerbate patients' subjective symptom experiences. Furthermore, this subtype is accompanied by higher levels of anxiety and depression and a lower perceived level of social support; both factors may be associated with a more pronounced subjective experience of physical symptoms, and there may be a mutually reinforcing relationship among these variables. This study identified substantial heterogeneity in PFS across postoperative cervical cancer patients. This finding provides evidence-based support for the targeted allocation of public health resources: patients with the “Multiple Coexisting Symptoms—Sleep Disturbance” subtype, accounting for 17.1% of the cohort, should be prioritized for intervention. This heterogeneity provides an empirical basis for future efforts to stratify follow-up assessment protocols by symptom cluster category. Future intervention studies could test whether category-specific monitoring schedules—rather than uniform assessment frequencies—improve the efficiency of clinical resource utilization and enable earlier identification of high-risk individuals.

To further validate the generalizability of our findings and compare them with symptom cluster studies in other cancers, we placed our results within a broader cross-cancer evidence spectrum. A study of the fatigue-pain-sleep disturbance-numbness/tingling symptom cluster in breast cancer survivors (39) showed that non-pharmacological interventions can effectively alleviate this cluster, and that a “low-symptom” or “mild-symptom” subtype is commonly present among breast cancer patients, with a proportion similar to the “Low Symptom Expression Type” identified in this study. A study (17) of gynecological cancer patients undergoing chemotherapy identified subtypes with varying symptom severity. The severe symptom subtype exhibited extremely high levels of pain, fatigue, and sleep disturbances, along with more severe comorbidities and lower quality of life—findings that closely align with the “Multisymptom Coexistence—Sleep Disorder Type” subtype identified in this study. Patients in this subtype represent a high-risk population with the heaviest symptom burden and an urgent need for prioritized clinical intervention. Among lung cancer patients, a latent profile analysis of the fatigue-pain-sleep disturbance symptom cluster similarly identified a subtype with a high symptom burden. Furthermore, the study (12) found that this symptom cluster is closely associated with metabolic dysregulation, suggesting that systemic inflammation and metabolic disorders may be common biological mechanisms underlying symptom clusters across cancer types. In contrast, postoperative cervical cancer patients may be more significantly affected by postoperative complications and psychological distress, factors that are rarely explored as core covariates in studies of other cancer types (40).

### 4.2 Factors influencing potential profiles of PFS in postoperative cervical cancer patients

#### 4.2.1 Common protective factors: perceived social support and laparoscopic surgery

This study found that perceived social support and laparoscopic surgery serve as common protective factors for the Pain-Dominated—Sleep-Impaired Type, the High Pain and Fatigue—Sleep Compensation Type, and the Multisymptom Coexistence—Sleep Disorder Type and exert a stabilizing effect across symptom clusters of varying severity. From a public health perspective, these two factors represent intervenable social determinants of health with broad protective effects across subtypes. Patients who have undergone cervical cancer surgery commonly experience significant psychological stress; higher levels of perceived social support are associated with positive emotions and greater psychological resilience (41). This association may coexist with greater psychological resilience in patients or may partially explain differences in symptom burden across subtypes. Factors related to social support—such as access to resources and family care—may be associated with a milder perception of physical discomfort (42). Patients with higher perceived levels of social support not only experience a reduced burden of daily activities through family care but also gain access to information on pain management methods and sleep improvement techniques, which in turn is associated with symptom relief. Laparoscopic surgery is associated with lower levels of postoperative pain (43); the reduced tissue damage resulting from minimally invasive surgery may be a potential mechanism underlying the milder symptom presentation in this group. These findings suggest that perceived social support may serve as a candidate indicator for future risk stratification models. Prospective studies are warranted to evaluate whether incorporating social support screening into admission assessments can effectively identify patients at elevated risk for moderate-to-severe symptom subtypes.

#### 4.2.2 Common risk factors: hospital-acquired anxiety and depression

The results of this study indicate that hospital-related anxiety and depression are common risk factors for the Pain-Dominated—Sleep-Impaired Type, the High Pain and Fatigue—Sleep Compensation Type, and the Multisymptom Coexistence—Sleep Disorder Types, with consistent associations observed across all symptom subtypes. The underlying mechanism may be that anxiety triggers neuroendocrine disturbances by activating the hypothalamic-pituitary-adrenal axis, thereby affecting pain thresholds and sleep rhythms (44). Patients with anxiety and depression often exhibit cognitive biases, focusing excessively on postoperative physical sensations and interpreting normal postoperative reactions as signs of worsening conditions, which further amplifies their subjective perception of symptoms. Studies (45) have confirmed that patients with anxiety and depression have higher pain scores and a higher incidence of sleep disturbances compared to those without emotional abnormalities, indicating the driving role of emotional factors in this symptom cluster. Given the consistent association between anxiety/depression and more severe symptom subtypes, psychological screening represents a promising component of future integrated symptom management models. The HADS or similar screening tools could be evaluated in future implementation research as a means to identify patients who may benefit from more intensive psycho-oncological support.

#### 4.2.3 Specific risk factors: monthly household income, age, place of residence, postoperative complications, and occupation

This study found that a monthly household income exceeding 5,000 yuan exerts a protective effect only on the High Pain and Fatigue—Sleep Compensation Type and Multisymptom Coexistence—Sleep Disorder Type. This suggests that the protective effect of economic factors on symptom clusters is dependent on symptom severity, and that the value of intervention is particularly pronounced for patients with moderate-to-severe symptom clusters. This reflects a healthy social gradient: the protective effect of economic resources only becomes fully apparent once the burden of symptoms reaches a certain level. This may be related to the fact that high-income patients typically have access to higher-quality care, specialized rehabilitation, and personalized nutritional support. A study (46) found that monthly household income indirectly moderates symptom cluster severity by influencing access to medical resources and psychological well-being. From the perspective of public health resource allocation, these findings indicate that financial status may serve as a potential stratification variable for future intervention designs. Future implementation research could test whether resource allocation strategies pecifically, prioritizing support for the most distressing symptom dimensions among low-income patients can effectively reduce the disparities in symptom burden associated with economic disadvantage.

This study found that age >40 years is a risk factor for the High Pain and Fatigue—Sleep Compensation Type and Multisymptom Coexistence—Sleep Disorder Type. Its negative impact does not affect all subtypes uniformly but primarily targets moderate-to-severe symptom clusters. This may be due to age-related declines in fibroblast proliferation and migration capacity, as well as reduced collagen synthesis, which in turn affect the quality and speed of wound healing (36). Additionally, middle-aged and older patients often have comorbidities such as hypertension and diabetes; their impaired vascular endothelial function and weakened immune status increase the risk of postoperative inflammatory responses and complications. Studies (47) show that the likelihood of fatigue increases with age. The finding that age >40 years was specifically associated with the two moderate-to-severe subtypes suggests that future perioperative risk stratification strategies may need to incorporate age as a key covariate. Further research could examine whether enhanced preoperative optimization of comorbidities—such as hypertension and diabetes—attenuates symptom burden in older patients following cervical cancer surgery.

The results of this study indicate that living in rural areas, postoperative complications, and unemployment are specific risk factors for Multisymptom Coexistence—Sleep Disorder Type. These three factors collectively point to the core issue of “social inequalities in health.” The impact in rural areas may be related to low coverage of postoperative rehabilitation guidance, a scarcity of continuity of care resources, and a lack of professional knowledge and skills regarding symptom management among patients and caregivers. Studies (48) show that satisfaction with postoperative symptom management among patients in rural areas is significantly lower than in urban areas, directly reflecting the impact of regional disparities on symptom clusters. Physical discomfort caused by postoperative complications, such as lymphedema and urinary retention, directly activates pain pathways, exacerbates feelings of fatigue, and severely disrupts normal sleep cycles. A study (49) confirms that postoperative complications following cervical cancer surgery are closely associated with a higher symptom burden and the co-occurrence of multiple symptoms among patients. The impact of unemployment may stem from patients' lack of stable income and the heavy financial burden of medical expenses, potentially leading them to forgo necessary interventions—such as analgesic medications and rehabilitation therapy—due to cost constraints. Additionally, unemployment undermines a sense of self-worth, triggering negative emotions such as loneliness and helplessness, which in turn affect patients' proactive ability to cope with symptoms. A study (50) found that unemployed cancer patients typically report a higher symptom burden and poorer health-related quality of life. The identification of rural residence, postoperative complications, and unemployment as subtype-specific risk factors highlights several candidate targets for future intervention design. First, rural residence—potentially reflecting limited access to continuity of care—suggests that future transitional care models might prioritize enhanced discharge planning and caregiver education for this population. Second, the strong association between postoperative complications and the most severe subtype indicates that prospective studies are needed to evaluate whether complication-triggered symptom monitoring protocols can mitigate the progression to multisymptom coexistence. Third, the link between unemployment and the severe subtype implies that financial distress may be an important mediator of symptom burden; future supportive care interventions could test whether incorporating financial navigation services into standard discharge planning improves symptom outcomes for unemployed or low-income patients.

## 5 Conclusion

Population heterogeneity in PFS among postoperative cervical cancer patients has been confirmed, with age, surgical approach, perceived social support, and anxiety and depression identified as key factors influencing symptom cluster classification. This provides a scientific basis for conducting individualized symptom assessments and early stratification of high-risk populations in clinical practice. This study has the following limitations. First, the sample was limited to cervical cancer patients within two months post-surgery at three Grade A Level 3 hospitals in Liaoning Province; patients from primary-level maternal and child health institutions and populations in other provinces were not included. Consequently, the four potential profile classifications identified in this study and the conclusions regarding the associations between risk factors and each subtype lack validation in independent external populations. The study results cannot be directly generalized to postoperative cervical cancer patient populations across medical institutions of different levels or in different regions nationwide. Second, as a cross-sectional study, this research collected only self-reported symptom data from patients at a single point in time, allowing only for the static identification of potential profile characteristics of symptom clusters in postoperative cervical cancer patients. The inability to conduct longitudinal validation makes it difficult to reveal the dynamic patterns of transition among the four symptom subtypes over the postoperative recovery period, as well as the long-term trends in the symptom burden associated with each subtype. Finally, although this study employed standardized scales for data collection, all symptom-related data relied on patients' subjective self-reports and lacked supporting evidence from objective physiological indicators. To address these limitations, a series of in-depth follow-up studies could be conducted to further refine the relevant conclusions and expand the research's value. First, expand the sample coverage to include patients from primary-level maternal and child health care institutions and conduct comparative studies between urban and rural populations to analyze differences in symptom clusters among cervical cancer patients across different levels of care and geographic regions, thereby enhancing the generalizability and extrapolation potential of the study conclusions; second, optimize the study design by conducting longitudinal follow-up surveys to collect dynamic data from patients at 1, 3, and 6 months post-surgery, and systematically analyze the temporal transition characteristics of PFS symptom subtypes and the long-term evolution of symptom burden; third, refine the research data and classification system by integrating objective physiological indicators with qualitative interview data to optimize the criteria for symptom cluster classification, thereby enhancing the scientific rigor and clinical utility of the classification results. Based on this, further exploration of the impact of different symptom subgroups on the efficiency of healthcare service utilization, medical costs, and long-term rehabilitation outcomes is expected to provide public health policymakers with more direct cost-benefit evidence, thereby promoting the inclusion of stratified symptom management in the scope of health insurance coverage for postoperative cancer rehabilitation.

## Data Availability

The raw data supporting the conclusions of this article will be made available by the authors, without undue reservation.
